# Chromatin Imbalance as the Vertex Between Fetal Valproate Syndrome and Chromatinopathies

**DOI:** 10.3389/fcell.2021.654467

**Published:** 2021-04-20

**Authors:** Chiara Parodi, Elisabetta Di Fede, Angela Peron, Ilaria Viganò, Paolo Grazioli, Silvia Castiglioni, Richard H. Finnell, Cristina Gervasini, Aglaia Vignoli, Valentina Massa

**Affiliations:** ^1^Department of Health Sciences, Università degli Studi di Milano, Milan, Italy; ^2^Human Pathology and Medical Genetics, ASST Santi Paolo e Carlo, San Paolo Hospital, Milan, Italy; ^3^Child Neuropsychiatry Unit–Epilepsy Center, Department of Health Sciences, San Paolo Hospital, ASST Santi Paolo e Carlo, Università degli Studi di Milano, Milan, Italy; ^4^Division of Medical Genetics, Department of Pediatrics, University of Utah School of Medicine, Salt Lake City, UT, United States; ^5^Departments of Molecular and Cellular Biology, Molecular and Human Genetics and Medicine, Center for Precision Environmental Health, Baylor College of Medicine, Houston, TX, United States; ^6^“Aldo Ravelli” Center for Neurotechnology and Experimental Brain Therapeutics, Università degli Studi di Milano, Milan, Italy

**Keywords:** fetal valproate syndrome, chromatinopathies, anti-epileptic drugs, neurodevelopment, HDAC inhibitor

## Abstract

Prenatal exposure to valproate (VPA), an antiepileptic drug, has been associated with fetal valproate spectrum disorders (FVSD), a clinical condition including congenital malformations, developmental delay, intellectual disability as well as autism spectrum disorder, together with a distinctive facial appearance. VPA is a known inhibitor of histone deacetylase which regulates the chromatin state. Interestingly, perturbations of this epigenetic balance are associated with chromatinopathies, a heterogeneous group of Mendelian disorders arising from mutations in components of the epigenetic machinery. Patients affected from these disorders display a plethora of clinical signs, mainly neurological deficits and intellectual disability, together with distinctive craniofacial dysmorphisms. Remarkably, critically examining the phenotype of FVSD and chromatinopathies, they shared several overlapping features that can be observed despite the different etiologies of these disorders, suggesting the possible existence of a common perturbed mechanism(s) during embryonic development.

## Introduction

Prenatal exposure to antiepileptic drugs (AEDs) are subject to the teratogenic effects associated with all of the frontline AED medications. Most women with epilepsy receiving adequate prenatal care will have uneventful pregnancies, but they are at a well-documented increased risk for having infants with congenital malformations compared to the general population (Viale et al., 2015). *In utero* AED exposure places their offspring at increased risk not only for major congenital malformations, but also for adverse neurological developmental outcomes. However, many of these risks can be mitigated through comprehensive prenatal maternal care by carefully selecting the type and dose of AEDs prior to conception and continuing to follow a proper therapeutic regimen throughout pregnancy (Tomson et al., 2011). Among all AEDs, valproate (2-propylpentanoic acid, VPA) exposure has been associated with the greatest risks of inducing severe teratogenicity (Lammer et al., 1987; Tomson et al., 2015). Several studies demonstrated a correlation between chronic exposure to VPA treatment and higher risk of displaying fetal anomalies–such as neural tube defects (NTDs), distinctive facial dysmorphia, craniofacial, and skeletal defects–in both in humans and in animal models (Massa et al., 2005, 2006). Among the teratogen-induced congenital malformations, the most commonly observed include spina bifida, atrial septal defects, cleft palate, hypospadias, polydactyly, and craniosynostosis (Macfarlane and Greenhalgh, 2018).

Animal experiments demonstrated morphogenic anomalies throughout the entire axial skeleton and vertebral transformations in rat embryos due to VPA exposure, suggesting a possible compromise of the expression of genes involved in vertebral segments development (Menegola et al., 1998, 1999). In addition, an altered serotonergic differentiation, which correlates with autism-like behavioral abnormalities, was observed both in rodent and zebrafish models in response to prenatal valproate exposure (Dufour-Rainfray et al., 2010; Jacob et al., 2014). The amount of fetal harm appears to be linked to the maternal concentration of the drug (Nau et al., 1981; Nau, 1985), especially when it occurs in the first trimester during fetal organogenesis (Macfarlane and Greenhalgh, 2018). In animal models, such as *Xenopus* and *Hyperolius*, the beginning of gastrulation was delayed up to neurulation upon embryonic exposure to VPA, and eventually they displayed NTDs of different types and degree (Oberemm and Kirschbaum, 1992). To date, the correlation between typical dysmorphic facial features and developmental outcomes is unclear (Kini et al., 2006; Nicolini and Fahnestock, 2018).

Fetal valproate syndrome (FVS, OMIM #609442) is a condition resulting from the therapeutic management of epileptic mothers with VPA during their pregnancy, and it is observed in up to 20–30% of children exposed to high VPA dosage *in utero* (Nau et al., 1991; Ornoy, 2009; Tomson et al., 2011). FVS is characterized by a constellation of congenital malformations and developmental delay, with patients displaying intellectual disability (ID) as well as autism spectrum disorder (ASD), and a distinctive facial appearance strikingly similar to the one described in genetic disorders known as chromatinopathies (DiLiberti et al., 1984). A new term “fetal valproate spectrum disorder” (FVSD) has recently been proposed to describe the range of clinical and developmental effects that are attributed to *in utero* VPA exposure (Clayton-Smith et al., 2019).

Valproate has been commonly used as an anti-seizure medication for over half a century (Meunier et al., 1963). Given its broad antiepileptic effect, it has also been clinically utilized as a mood stabilizer in the treatment of bipolar disorders and in other neurological conditions–i.e., migraine and neuropathic pain, exposing many more women of reproductive age to this medication (Johannessen and Johannessen, 2003). In addition, this antiepileptic drug has shown anticancer properties for several tumors (Shah and Stonier, 2019), and its use in combination regimens with cytotoxic chemotherapy seems to be promising (Brodie and Brandes, 2014). VPA is also known to be a potent histone deacetylase inhibitor (HDACi) and therefore acts on chromatin. It is known to have dose-related teratogenic properties resulting in altered gene expression and potent inhibition of the histone deacetylases (HDAC) enzymes family (Schölz et al., 2015). Among the various hypotheses that have been proposed for the teratogenicity of VPA, its HDACi effects that is believed to be represent the principle underlying teratogenic mechanism. VPA’s anti-seizure activity can also be explained by its ability to modulate gene expression through the inhibition of HDAC enzymes (Göttlicher et al., 2001; Jacob et al., 2013; Brunton et al., 2018).

Valproate perturbs the cell’s epigenetic machinery controlling its chromatin state. In this context, a group of heterogeneous genetic disorders known as the chromatinopathies, are believed to be caused by mutations in genes that regulate the conformation and function of chromatin, thus acting in concert with epigenetic mechanisms. Defects in the functional network between the complexes associated with chromatin could lead to alterations in gene expression and protein function. As estimated, there are over 80 Mendelian diseases associated with incorrect functioning of the “epigenetic machinery”, the majority of which presents with neurological defects and ID (Fahrner and Bjornsson, 2019). Kabuki syndrome (OMIM #147920 and #300867) (Niikawa et al., 1981) and CHARGE syndrome (OMIM #214800) (Pagon et al., 1981) are among the most well-known and studied chromatinopathies, for the cascading effect of the causative genes on different cell pathways. These syndromes are associated with ID and distinctive craniofacial dysmorphisms that are pathognomonic.

In this review, we explore shared features between FVSD and selected chromatinopathies, leading us to the hypothesis that these disorders, despite divergent etiologies (i.e., environmental or genetic), could operate through a common perturbed mechanism during embryonic development. As such, VPA-induced FVSD is a phenocopy of select chromatinopathies.

## Valproate Mechanism of Action

Valproate has multiple cellular mechanisms of action consistent with its broad clinical efficacy. This compound appears to suppress repetitive high-frequency neuronal focus by blocking voltage-dependent sodium channels, but at sites that are different from other AEDs. VPA also appears to increase GABA concentrations in the brain at clinically relevant doses, without having direct effects on the GABA (A) receptors, potentiated by a presynaptic effect of valproate on GABA (B) receptors. In addition, VPA can increase GABA synthesis by activating the enzyme glutamic acid decarboxylase (GAD).

The molecular mechanisms underlying FVSD have not been fully established, although the consequences of *in utero* VPA exposure have been investigated for several decades. Such effects include apoptotic neurodegeneration observed in the developing rat brain (Bittigau et al., 2002), enhanced synaptic plasticity exhibited in the rat medial prefrontal cortex (Sui and Chen, 2012), and a decrease in folic acid (Wegner and Nau, 1992), suggesting that inadequate embryonic and fetal antioxidant defense mechanisms and consequent oxidative stress could be responsible for brain damage secondary to VPA teratogenicity (Ornoy, 2009).

Despite the fact that VPA has been shown to be neuroprotective in neurons through *Bcl-2* upregulation (Chen et al., 1999), its administration in critical developmental stages causes morphological defects and impaired social behavior in rats (Kim et al., 2011). *In utero* VPA exposure in mouse pups on gestational day 11 leads to dysfunctional pre-weaning social behavior, together with delayed development, impaired olfactory discrimination and reduced cortical *Bdnf* expression, suggesting that VPA-driven perturbations in neuronal plasticity may underlie the behavioral phenotype (Roullet et al., 2010). Similar to the results of VPA exposure of pregnant rats, neural progenitor cells (NPCs) of murine embryos exposed on gestational day E12 showed a reduced apoptotic cell death, which is fundamental to the proper regulation of NPCs during a developmentally critical period, suggesting another possible mechanism underlying FVSD defects (Go et al., 2011).

Alterations in embryonic gene expression following VPA exposure appears to be one of the primary mechanisms underlying VPA’s teratogenicity. Previous studies showed that VPA alters Wnt signaling by inducing Wnt-dependent gene expression at doses that cause developmental effects (Phiel et al., 2001; Wiltse, 2005). This is due to its role as an HDAC inhibitor, which consists of deregulating class I HDACs, thus counteracting their normal activity of histone acetylation marks removal. This action induces chromatin changes converting segments of heterochromatin into euchromatin. VPA exposure can lead to hyperacetylation of histones and following activation of genes related to cell cycle and apoptosis, possibly explaining its teratogenic action (Göttlicher et al., 2001). For instance, hyperacetylation of all *Hoxb* developmental genes has been observed in mouse embryonic stem cells exposed to VPA, with increased levels of H3K9ac at upstream, promoter and coding regions across the entire *Hoxb* cluster (Boudadi et al., 2013).

Previous studies showed how HDACi is involved in early neuronal processes: exposure of neurulation-stage mouse embryos to VPA can cause NTDs and skeletal malformations (Finnell et al., 2002), supported by *in vivo* studies on chick embryos in which a complete failure of neural tube closure occurred (Murko et al., 2013). Since VPA exposure alters gene expression in the somitic tissues of mouse embryos (Massa et al., 2005) and an increased histone H4 acetylation in the caudal neural tube was observed, modulation of acetylation was hypothesized as mediating the effect of VPA on neurulation (Massa et al., 2005, 2009; Menegola et al., 2005).

## Valproate in Clinical Practice

Valproate is a wide-spectrum anti-seizure medication that can be used to treat almost all types of seizure disorders (tonic clonic seizures, absence seizures, myoclonic seizures, less frequently in clonic seizures, tonic seizures and atonic seizures) (Perucca, 2002). It is used as first-line antiepileptic drug in generalized seizures; VPA may also be used in focal seizures, although it is no longer the first choice of neurologists (Tomson et al., 2015).

Valproate is also a mood stabilizer that is used in the treatment of bipolar disorders and other psychiatric conditions, including: anxiety disorders, post-traumatic stress disorder, substance abuse, and schizophrenia. VPA also appears to be an effective treatment for tardive dyskinesia thanks to its GABA-potentiating properties (Swann et al., 2002), for migraine prophylaxis, and for the treatment of neuropathic pain, in particular for trigeminal neuralgia (Johannessen and Johannessen, 2003).

Typically, the initial dose of oral valproate is 10–15 mg/kg per day. If necessary, the dose can be increased with weekly increments of 5–10 mg/kg up to a maximum dose of 60 mg/kg/day. It is recommended to monitor VPA blood level during treatment, as well as blood count, liver enzymes, and coagulation tests, in order to avoid any potential side effects of the drug.

Valproate can alter vitamin D metabolism and affect bone mineral density, therefore 25-hydroxyvitamin D levels should be monitored. It may be useful to obtain serum amylase and lipase levels in cases where symptoms suggestive of pancreatitis, such as abdominal pain, nausea, vomiting and anorexia have occurred. Furthermore, ammonium levels should be monitored in patients receiving VPA who exhibit signs of vomiting or lethargy as the treatment inhibits *N*-acetyl glutamate, leading to systemic disruption and hyperammonemia (Bruni et al., 1979; Batshaw and Brusilow, 1982; Asconapé et al., 1993; Patsalos et al., 2008).

It is strongly recommended that VPA administration should be avoided during pregnancy; however, if necessary, a slow-release formulation that limits peak concentrations of the drug using the lowest efficacious dose possible should be given along with the administration of a high dose of folic acid. While folic acid has not be shown to be effective in reducing the prevalence of NTDs, it has been shown to be protective in limiting adverse cognitive consequences of VPA treatment, especially with respect to language skills (Meador et al., 2020).

## Clinical Features Associated With FVSD

Studies conducted by Robert and Guibaud (1982) first drew attention to the increased risk of spina bifida after exposure to VPA in pregnancy. Subsequently, the initial reports of children suffering from FVSD were published (DiLiberti et al., 1984). FVSD is characterized by major and minor malformations, facial dysmorphia and impaired development with particular risks related to NTDs (Lindhout and Schmidt, 1986), congenital heart disease, ophthalmological, (Glover et al., 2002) and genitourinary abnormalities (DiLiberti et al., 1984; Ozkan et al., 2011), cleft palate (Jackson et al., 2016), overlapping fingers, and scalp defects (DiLiberti et al., 1984; Clayton-Smith and Donnai, 1995; Clayton-Smith et al., 2019).

Neurological development is impaired in many affected individuals. An increased risk of attention deficit hyperactivity disorder (ADHD) and ASD is often observed in these patients (Bromley et al., 2013, 2014, 2019; Christensen et al., 2013).

**FIGURE 1:**
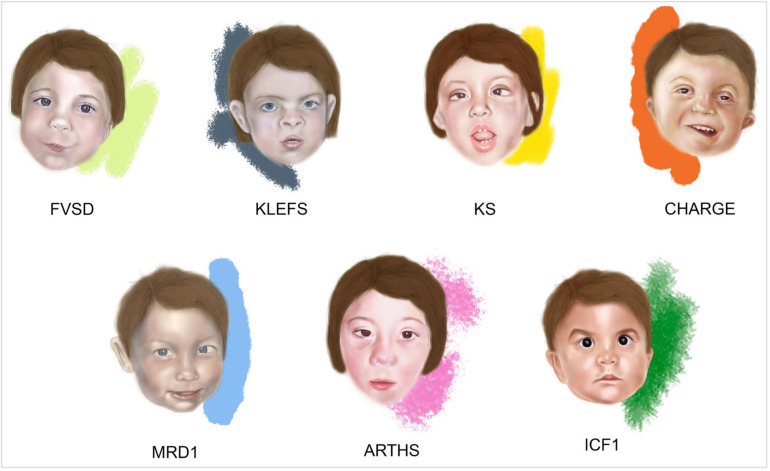
*Facies* of fetal valproate spectrum disorders (FVSD) and related overlapping chromatinopathies. Distinctive facial phenotypes of patients affected by FVSD (Schorry et al., 2005), KLEFS (Willemsen et al., 2012), KS (Makrythanasis et al., 2013), CHARGE (Hefner and Fassi, 2017), MRD1 (Talkowski et al., 2011), ARTHS (Kennedy et al., 2019), and ICF1 (Gössling et al., 2017).

The typical facial features of FVSD include: swelling of the metopic suture, highly-arched eyebrows, hypertelorism, wide nasal bridge, short nose with anteverted nostrils, small mouth with thin upper lip and flat filament of the inverted lower lip (Ardinger et al., 1988; Clayton-Smith and Donnai, 1995; Kozma, 2001; Schorry et al., 2005; Kini et al., 2006; Chandane and Shah, 2014; Mohd Yunos and Green, 2018) (Figure 1).

## Differential Diagnoses of FVSD

Fetal valproate spectrum disorder diagnosis has been challenging in many ways, from gathering correct information about prenatal VPA exposure, to obtaining a comprehensive grasp of the clinically varied diagnostic phenotypic signs. Indeed, clinical presentations of affected patients show variability and the prevalence of neurocognitive dysfunction is higher than the prevalence of structural malformations, complicating the path toward reliable diagnosis (Clayton-Smith et al., 2019). In fact, not all the FVSD individuals even display dysmorphisms, which can be age dependent and rather subtle, thus recognizable only by experienced dysmorphologists. When FVSD signs are ascertained, physicians are often challenged by overlapping phenotypes associated with the following syndromes (Figure 1 and Table 1).

**TABLE 1:** Fetal valproate spectrum disorders (FVSD) clinical signs in chromatinopathies.

FVSD clinical signs	KLEFS	KS	CHARGE	MRD1	ARTHS	ICF
**Facial dysmorphisms**						
*Scalp defects*	**−**	**−**	**−**	**−**	**−**	**−**
*High/prominent forehead*	**+**	**−**	**+**	Broad	**−**	±
*Bitemporal narrowing*	**−**	**−**	±	**−**	**+**	**−**
*Arched eyebrows*	**+**	**+**	**−**	+	**−**	±
*Hypertelorism*	**+**	±	±	**−**	**−**	+
*Epicanthal folds*	**+**	**−**	+	**−**	±	+
*Ears abnormalities*	**+**	+	+	+	±	+
*Midface hypoplasia*	**+**	**−**	+	±	**−**	**−**
*Short nose*	**+**	**−**	±	+	**−**	±
*Broad/flat nasal bridge*	**+**	+	**−**	+	Broad tip	+
*Anteverted nostrils*	**+**	**−**	**−**	**−**	**−**	±
*Long smooth philtrum*	**+**	**−**	**−**	**−**	**−**	**−**
*Small mouth*	**−**	**−**	**−**	**−**	**−**	**−**
*Thin upper lip*	**−**	**−**	±	+	+	**−**
*Downturned corners of the mouth*	+		**−**	+	±	**−**
**Congenital anomalies**						
*Cleft palate*	+	+	+	±	±	±
*Macroglossia*	**−**	**−**	+	**−**	**−**	±
*Micro/retrognathia*	**−**	+	+	+	±	±
*Microcephaly*	+	+	+	+	+	na
*Trigonocephaly*	**−**	**−**	**−**	**−**	**−**	**−**
*Brachycephaly*	+	**−**	**−**	+	±	**−**
**Other malformations**						
*Neural tube or CNS defects*	+	+	+	**−**	±	±
*Ophthalmological defects*	±	+	+	±	+	**−**
*Muscoskeletal anomalies*	**−**	+	+	+	±	**−**
*Congenital heart defects*	+	+	+	+	+	±
*Genitourinary anomalies*	+	+	+	±	±	**−**
Developmental delay	+	+	+	+	+	±
Intellectual disability	+	+	+	+	+	±
Speech delay	+	+	+	+	+	+
Behavioral problems	+	±	+	+	+	**−**
**References**						
DiLiberti et al., 1984; Ardinger et al., 1988; Kozma, 2001; Schorry et al., 2005; Kini et al., 2006; Chandane and Shah, 2014; Mohd Yunos and Green, 2018	Willemsen et al., 2012; Hadzsiev et al., 2016; Arora et al., 2018	Makrythanasis et al., 2013; Adam et al., 2019; Shangguan et al., 2019	Hefner and Fassi, 2017; van Ravenswaaij-Arts and Martin, 2017	Van Bon et al., 2010; Talkowski et al., 2011; Hodge et al., 2014; Mullegama et al., 2016	Arboleda et al., 2015; Tham et al., 2015; Millan et al., 2016; Murray et al., 2017; Kennedy et al., 2019	Hagleitner et al., 2008; Weemaes et al., 2013; van den Boogaard et al., 2017; Kamae et al., 2018

*KLEFS, Kleefstra syndrome; KS, Kabuki syndrome; CHARGE, CHARGE syndrome; MRD1, mental retardation autosomal dominant 1; ARTHS, Arboleda-Tham syndrome; ICF1, immunodeficiency, centromeric instability, and facial anomalies syndrome 1; +, present; −, absent; ±, present in few cases; na, not available.*

### Kleefstra Syndrome

Kleefstra syndrome (KLEFS, OMIM #610253, #617768) is a rare condition characterized by heterozygous genomic deletions at chromosome 9q34.3 removing the *EHMT1* gene or *EHMT1* point mutations (KLEFS1), or pathogenic variants in *KMT2C* on chromosome 7q36.1 (KLEFS2), mostly *de novo*. *EHMT1* and *KMT2C* genes encode two histone methyltransferases. The prevalence is estimated to be 1:120,000 individuals affected by neurodevelopmental disorders. Patients with Kleefstra syndrome exhibit a distinctive phenotype including hypotonia; major anomalies such as congenital heart defects and genitourinary abnormalities; behavioral and developmental manifestations with ID of variable severity, and in some cases severe speech delay. Typical facial dysmorphisms include: microcephaly, arched or straight with synophrys eyebrows, mildly up-slanted palpebral fissures, hypertelorism, short nose with anteverted nares and bulbous nasal tip, thick mouth, and everted lower lip (Kleefstra and De Leeuw, 1993; Kleefstra et al., 2006, 2012; Koemans et al., 2017) (Figure 1).

Fetal valproate spectrum disorders has been recently defined as a “phenocopy” of Kleefstra syndrome by Arora et al. (2018). Despite a preliminary diagnosis of FVSD–due to maternal intake of VPA during pregnancy and with clear facial characteristics that are typically attributable to FVSD–a more thorough examination of the facial features revealed subtle differences. Specific features of the proband included the presence of a broad forehead and brachycephaly in a child with FVSD, who had cephalic deformation due to the premature fusion of the metopic suture, scattered eyebrows, and pointed chin. Genetic testing revealed a *de novo* deletion on 9q34.3 that is known to cause Kleefstra syndrome. The convergent mechanism present in both conditions is their role in epigenetic modulation that mediates the modification (acetylation, methylation, etc.) of histone proteins and DNA demethylation, which might be responsible for the overlapping phenotype of FVSD and Kleefstra Syndrome (Willemsen et al., 2012; Hadzsiev et al., 2016; Arora et al., 2018) (Table 1).

### Kabuki Syndrome

Specific dysmorphisms, postnatal growth delay, skeletal anomalies, and ID are typical features of another chromatinopathy such as Kabuki syndrome (KS, OMIM #147920 and #300867) (Kuroki et al., 1981; Niikawa et al., 1981; Adam et al., 2019). KS is caused by heterozygous pathogenic variants in *KMT2D* or *KDM6A* genes (Ng et al., 2010; Banka et al., 2012; Lederer et al., 2012; Miyake et al., 2013), on chromosome 12q13.12 and Xp11.3, causing KS1 and KS2, respectively. These genes altered in KS encode for a histone methyltransferase and a histone demethylase exerting their effect on different histone residues that favor the opening of chromatin and leading to the same downstream effects on gene expression, ultimately resulting in the same condition. Aside from ID, developmental impairment and congenital heart defects, KS shares with FVSD specific craniofacial features such as arched eyebrows, wide nasal bridge, and cleft palate (Makrythanasis et al., 2013; Adam et al., 2019; Shangguan et al., 2019) (Figure 1 and Table 1).

### CHARGE Syndrome

CHARGE syndrome (OMIM #214800) is an acronym that summarizes the main clinical manifestations, namely Coloboma of the eye, Heart defect, choanal Atresia, Retardation of psychomotor development and growth, Genital hypoplasia, and Ear abnormalities. This syndrome is caused by heterozygous pathogenic variants in *CHD7* (OMIM # 608892), encoding an epigenetic regulator that is involved in the ATP-dependent remodeling of chromatin (Vissers et al., 2004; van Ravenswaaij-Arts and Martin, 2017). Interestingly, Shah et al. (2014) and Jackson et al. (2014) reported on five children affected by FVSD exhibiting unilateral or bilateral ocular coloboma, one of the main manifestations of CHARGE syndrome. Indeed, VPA acting as a HDAC inhibitor reduces the expression of *PAX2* and *PAX6*, which are implicated in ocular development (Pennati et al., 2001; Balmer et al., 2012). Of note, CHARGE syndrome shares autism-like disturbances, congenital anomalies and malformations together with specific facial features with FVSD (Hefner and Fassi, 2017; van Ravenswaaij-Arts and Martin, 2017) (Figure 1 and Table 1).

### Mental Retardation Autosomal Dominant 1

Mental retardation autosomal dominant 1 (*MRD1*, OMIM #156200) or *MBD5* haploinsufficiency is a neurodevelopmental disorder caused by heterozygous variants in *MBD5* or a deletion encompassing all or part of this gene sequence on chromosome 2q23.1 (Vissers et al., 2003; Talkowski et al., 2011). *MBD5* encodes for a methyl-CpG-binding domain protein. MBD5 is part a class of proteins that bind to DNA with a transcriptional repressor activity. In Camarena et al. (2014) MBD5 was shown to act as transcriptional activator *in vitro.* Hence, MBD5 is considered a “reader” of the epigenetic machinery (Camarena et al., 2014). Patients display ID and developmental delay, sleep disturbances, seizures, severe speech impairment, behavioral problems, feeding difficulties, congenital anomalies mainly affecting the skeletal and cardiovascular systems, and dysmorphic signs. Among them, *MRD1* is characterized by broad forehead, highly arched eyebrows, outer ear abnormalities, short nose with broad nasal bridge, thin upper lip, and downturned mouth angles, which are remarkably overlapping with FVSD (Van Bon et al., 2010; Talkowski et al., 2011; Hodge et al., 2014; Mullegama et al., 2016) (Figure 1 and Table 1).

### Arboleda-Tham Syndrome

Pathogenetic variants in the *KAT6A* gene, located on chromosome 8p11.21 cause Arboleda-Tham syndrome (ARTHS, OMIM #616268) or Mental retardation autosomal dominant 32 (MRD32), a recently described disorder affecting neurodevelopment and associated with ID (Arboleda et al., 2015; Tham et al., 2015). KAT6A is a lysine-acetyltransferase involved in chromatin opening, transcriptional regulation, cellular replication and therefore, in multiple developmental programs (Voss et al., 2009). Kennedy et al. (2019) extensively described phenotypes of novel and previously reported ARTHS patients, who display distinctive clinical signs such as ID, developmental and speech delay, cardiac and ophthalmological defects, gastrointestinal problems, sleep disturbance, autism-like behavior and typical dysmorphisms (Arboleda et al., 2015; Tham et al., 2015; Millan et al., 2016; Murray et al., 2017), many of them overlapping with the FVSD phenotype (Figure 1 and Table 1).

### Immunodeficiency, Centromeric Instability and Facial Anomalies Syndrome

Immunodeficiency, centromeric instability and facial anomalies syndrome 1 (ICF1, #OMIM 602900) is a rare autosomal recessive disorder characterized by hypogammaglobulinemia leading to severe recurrent infections, instability of pericentromeric regions of chromosomes 1, 9, and 16 in mitogen-stimulated lymphocytes, and facial dysmorphisms (Maraschio et al., 1988; Ehrlich et al., 2006). When the mapping of a locus associated to ICF syndrome on chromosome 20 was performed in 1998 (Wijmenga et al., 1998), pathogenic variants in *de novo* DNA methyltransferase gene *DNMT3B* were identified, occurring in about half of ICF patients (Hansen et al., 1999; Xu et al., 1999). DNMT3B is involved in the establishment of DNA methylation patterns in early life and during cell differentiation. Hypomethylation of pericentromeric satellite 2 and 3 repeats represents the molecular hallmark of ICF syndrome (Jeanpierre et al., 1993), making it the first human disorder linked to a constitutive defect in DNA methylation. In addition to distinctive signs such as immunoglobulin deficiency and consequent recurrent infections (mainly respiratory and gastrointestinal), ICF1 patients also display some features that are common to FVSD: hypertelorism, epicanthus, flat nasal bridge, macroglossia, micrognathia, low-set ears, speech, and developmental delay, and–in a minority of affected individuals–CNS anomalies, congenital heart defects and ID (Hagleitner et al., 2008; Weemaes et al., 2013; van den Boogaard et al., 2017; Kamae et al., 2018) (Figure 1 and Table 1).

### Other Genetic Disorders

Qiao et al. (2019) recently described a 19 years-old man with ID and distinctive facial features who had a clinical diagnosis of FVSD and was later found to carry a *de novo* pathogenic variant in the *PURA* gene on chromosome 5q31. PURA-related neurodevelopmental disorders include Mental Retardation autosomal Dominant 31 (MRD31, #OMIM 616158) or PURA syndrome, caused by heterozygous mutations in the *PURA* gene or a 5q31.3 deletion affecting completely or partially eliminating the *PURA* sequence (Brown et al., 2013; Hunt et al., 2014; Lalani et al., 2014; Tanaka et al., 2015). This causative gene encodes for a DNA- and RNA-binding protein critical for survival and development of mammalian hematopoietic and central nervous systems (Daniel and Johnson, 2018). Shared phenotypic features between PURA disorders and FVSD are ID and developmental delay, heart defects, urinary and ophthalmological abnormalities, and distinctive facial dysmorphism such as high/broad forehead, hypertelorism, wide nasal bridge, and thin upper lip (Reijnders et al., 2018). Furthermore, fetal valproate exposure has been reported to cause other malformation complexes such as Baller-Gerold syndrome (BGS, OMIM #218600), an ultra-rare disorder caused by pathogenic variants in the *RECQL4* gene on chromosome 8p24, and inherited in an autosomal recessive manner (Baller, 1950; Gerold, 1959). BGS patients display a plethora of phenotypic features (Van Maldergem et al., 1992), some of which are overlapping with FVSD. These include: ID, developmental delay, limb and congenital heart defects, genitourinary anomalies and facial dysmorphisms (Iype et al., 2008). Mutations in the *RECQL4* gene that codes for an ATP-dependent DNA helicase essential for genome integrity and involved in DNA replication, recombination and repair (Bachrati and Hickson, 2008) have also been reported in Rothmund-Thomson (RTS, OMIM #268400) families (Kitao et al., 1999). In particular, children affected with type II RTS share a variety of clinical features with BGS patients (Rothmund, 1868; Thomson, 1936; Megarbane et al., 2000; Van Maldergem et al., 2006; Larizza et al., 2010). Considering the similarities among BGS and RTS patients, there are multiple overlapping features with FVSD that can be observed–e.g., head and nose dysmorphisms, developmental delay, cardiac defects and skeletal anomalies. Although these neurodevelopmental disorders are not considered chromatinopathies, it is worthy of note that PURA and RECQL4 are transcriptional regulators, and helicases are considered a guardian of the genome, such that they are involved in proper chromatin maintenance.

## Shared Epigenetic and Gene Expression Alterations

Gene expression deregulation by VPA has been widely investigated over the last few decades (Marchion et al., 2005; Jergil et al., 2009, 2011; Chiu et al., 2013; Shinde et al., 2016; Balasubramanian et al., 2019; Kotajima-Murakami et al., 2019; Lin et al., 2019; Sanaei and Kavoosi, 2019). Interestingly, some of the causative genes of the aforementioned syndromes are dysregulated in different experimental models. *Ehmt1* was found to be downregulated in brains of mice exposed to VPA *in utero*, *Kdm6a*, and *Dnmt3b* appeared to be upregulated in the same model (Kotajima-Murakami et al., 2019), while *Chd7* was downregulated in embryonal carcinoma cells upon VPA exposure (Jergil et al., 2009). In addition, *Ehmt1* and its human orthologs were downregulated in neural stem/progenitor cells of a mouse model for KS1 (Carosso et al., 2019) and in lymphoblastoid cell lines derived from 2q23.1 deletion syndrome patients (Mullegama et al., 2016), respectively. Expression of *DNMT3B* was decreased in iPSCs derived from KS1 patient (Carosso et al., 2019). Furthermore, *Kdm6a* and *Chd7* have been reported to be interlinked in terms of gene expression regulation (Mansour et al., 2012; Hsu et al., 2020), and in a mouse model expressing catalytically inactive *Dnmt3b*, they share opposite behavior (Lopusna et al., 2021).

In Table 2, shared pathways with genes deregulated by VPA and downregulated in models of causative genes for KLEFS, KS, CHARGE, MRD1, and ARTHS or ICF1 are summarized (Katsumoto et al., 2006; Issaeva et al., 2007; Min et al., 2007; Fan, 2008; Gupta-Agarwal et al., 2012; Mansour et al., 2012; Balemans et al., 2014; Chen et al., 2014; Kim et al., 2014; Schulz et al., 2014; Turner-Ivey et al., 2014; Gigek et al., 2015; Dhar et al., 2016, 2018; Fang et al., 2016; Mullegama et al., 2016; Sheikh et al., 2016, 2017; Feng et al., 2017a, b; Shpargel et al., 2017; Whittaker et al., 2017; Baell et al., 2018; Marie et al., 2018; Yao et al., 2018, 2020; Carosso et al., 2019; Machado et al., 2019; Nowialis et al., 2019; Cieslar-Pobuda et al., 2020; Frega et al., 2020; Hsu et al., 2020; Kong et al., 2020; Liu et al., 2020; Xu et al., 2020; Ying et al., 2020; Fei et al., 2021; Lopusna et al., 2021). Of note, the most commonly shared pathways involve either morphogenesis signals (for example, beta1 integrin cell surface interactions and extracellular matrix organization), or possible defects of the central nervous system (such as axon guidance and neuronal system). As such, given the recent description of ARTHS, it would be interesting to reassess this matter in the future utilizing state of the art molecular studies.

**TABLE 2:** Shared pathways between FVSD and chromatinopathies.

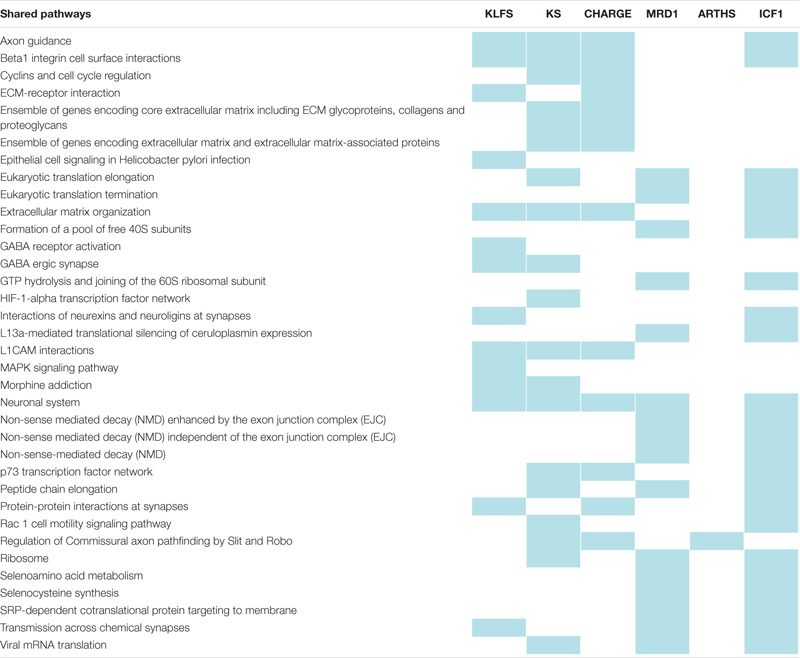

*KLEFS, Kleefstra syndrome; KS, Kabuki syndrome; CHARGE, CHARGE syndrome; MRD1, mental retardation autosomal dominant 1; ARTHS, Arboleda-Tham syndrome; ICF1, immunodeficiency, centromeric instability, and facial anomalies syndrome 1.*

## Conclusion

It is well established that the mammalian epigenome can change during embryonic development and be influenced by genetic and/or environmental factors, even though some molecular mechanisms underlying these modifications are yet not clear (Finnell et al., 2002; Xu and Xie, 2018). Chromatinopathies represent a heterogeneous group of Mendelian disorders with defects in the epigenetic apparatus, leading to an imbalance in the chromatin state and consequent aberrant gene expression. As described above, these disorders share several overlapping clinical signs, though with some specific features allowing dysmorphologists to recognize each individual syndrome. We highlighted similarities between the discussed chromatinopathies and FVSD, pointing out shared features in these genetic- and teratogen-induced disorders. As reported in Table 1, overlapping clinical signs are primarily ID and developmental delay, present in 5 out of 5 chromatinopathies described herein (6/6), speech delay (6/6), ASD-like behavior (5/6), microcephaly (5/6), cardiac (6/6) and ophthalmological defects with different degree of severity (5/6), cleft palate (6/6), musculoskeletal anomalies (4/6), and dysmorphic features such as highly arched or thick eyebrows (4/6) and ears abnormalities (6/6).

Intriguingly, despite the different etiology of a FVSD and the chromatinopathies, the action of VPA–i.e., an HDACi acting on chromatin–can suggest a similar pathogenetic mechanism common to the other rare genetic disorders, giving rise to the observed shared phenotypic signs. Furthermore, recent work showed that recognition of FVSD *facies* can identify individuals with high risk of cognitive deficits, independently of VPA exposure and even in the absence of major malformations (Bromley et al., 2019). Taken together, these pieces of evidence support the hypothesis that FVSD may be considered as a phenocopy of chromatinopathy, caused in this case by environmental factors, and that a further investigation of this aspect could help elucidate the correlation between typical congenital anomalies and neurodevelopment.

## Author Contributions

CG, AV, and VM conceived the manuscript. CP and EDF wrote the manuscript. AP reviewed clinical information. IV wrote sections of the manuscript. PG and SC read and edited the manuscript. RHF reviewed and revised the manuscript. All authors contributed to manuscript revision, they have all read and approved the manuscript.

## Conflict of Interest

RF formerly held a leadership position in TeratOmic Consulting LLC. This now dissolved organization provided expert consulting support in birth defects litigation. The remaining authors declare that the research was conducted in the absence of any commercial or financial relationships that could be construed as a potential conflict of interest.
